# Conformational Antibody Binding to a Native, Cell-Free Expressed GPCR in Block Copolymer Membranes

**DOI:** 10.1371/journal.pone.0110847

**Published:** 2014-10-20

**Authors:** Hans-Peter M. de Hoog, Esther M. Lin JieRong, Sourabh Banerjee, Fabien M. Décaillot, Madhavan Nallani

**Affiliations:** 1 ACM Biolabs Pte Ltd, Research Techno Plaza XF-6, Singapore, Singapore; 2 Singapore Immunology Network, Agency for Science, Technology and Research (A*STAR), Singapore, Singapore; 3 Centre for Biomimetic Sensor Science, School of Materials Science and Engineering, Nanyang Technological University, Singapore, Singapore; Cleveland Clinic Lerner Research Institute, United States of America

## Abstract

G-protein coupled receptors (GPCRs) play a key role in physiological processes and are attractive drug targets. Their biophysical characterization is, however, highly challenging because of their innate instability outside a stabilizing membrane and the difficulty of finding a suitable expression system. We here show the cell-free expression of a GPCR, CXCR4, and its direct embedding in diblock copolymer membranes. The polymer-stabilized CXCR4 is readily immobilized onto biosensor chips for label-free binding analysis. Kinetic characterization using a conformationally sensitive antibody shows the receptor to exist in the correctly folded conformation, showing binding behaviour that is commensurate with heterologously expressed CXCR4.

## Introduction

G protein-coupled receptors (GPCRs) are cell-surface receptors that mediate the communication of the cell with its environment and, as such, form important targets for therapeutic intervention. GPCRs are notoriously hard to obtain in a format amenable to biophysical studies, which depending on the characterization method, requires moderately to highly pure receptor preparations. The limited success of obtaining sufficiently pure receptor preparations results from low levels of expression of the native proteins and their low stability in lipid membranes. To boost expression levels, researchers have resorted to the use of engineered cell lines (e.g., HEK, CHO, Sf9 cells) as well as engineered proteins, which may involve mutagenesis, deletion of destabilizing sequence elements, and production of GPCR chimeras. [1] In a few instances, this has resulted in comparatively stable receptors that can be expressed at high concentrations and are even amenable to crystallization although the effects of such types of protein engineering on the native function of these intricate proteins remains a topic of debate. [2], [3] More importantly, these methods are highly labor and time intensive and there is currently no method to quickly produce stable receptor preparations in amounts suitable for thorough physical characterization.

A second, comparatively unexplored, approach would be to engineer the membrane surrounding the protein, in order to exceed the limited physical stability afforded to it by a lipid bilayer.[4]–[7] Our research, along with others, has shown that membrane proteins can insert into the fully synthetic membrane of block copolymer vesicles or polymersomes,[8]–[10] whose bilayer architecture is akin to that of the plasma membrane but with unprecedented physical stability. [11] Extension of this concept to include cell-free expression of the protein allows direct insertion of the native, full-length membrane proteins into the polymer membrane, as we have shown qualitatively in the past for the D2 receptor. [12], [13].

We here show evidence that, at least for the GPCR currently under study, this ‘artificial cell membrane’ (ACMs), when subjected to cell-free synthesis, presents properly folded, native protein and exhibit sufficient stability to allow label-free biosensor analysis. As the GPCR of choice we employ CXCR4, a relatively well-characterized chemokine receptor and a key target for immunological intervention as well as a co-receptor for entry of HIV into T cells. [14] The native CXCR4 is expressed and inserted into polybutadiene-*b*-poly(ethylene oxide) polymersomes, and immobilized on a surface-plasmon resonance (SPR) biosensor chip surface for analysis of the binding characteristics of antibodies specific (Ab) to CXCR4.[15]–[17] Screening of the CXCR4-ACMS against a conformationally sensitive Ab, as well as its natural ligand SDF-1 shows that such in-vitro expressed receptors exist in the correctly folded conformation. From a fundamental point of view the presented approach highlights the role of the polymer membrane in extending stability to CXCR4, and suggests that membrane engineering could form a viable alternative to protein engineering for exploring the biophysics of this class of receptors.

## Materials and Methods

For a detailed description of the materials used, preparation of the polymersomes, cloning and in-vitro expression of CXCR4, as well as radioligand binding assays and melting curves, see the supporting information.

### Biosensor analysis

All experiments were performed on a Biacore T200 (GE healthcare).

To capture the streptavidin on the Biacore AU chip (GE healthcare, UK), a thoroughly cleaned Au chip was first functionalized with 11-mercaptoundecanoic acid (0.1 M in ethanol). The surface was then activated with a mixture of 0.2 M 1-ethyl-3-(3-dimethylaminopropyl) carbodiimide (EDC) and 0.05 M *N*-hydroxysuccinimide (NHS) at a flow rate of 10 µL min^−1^ for 7 min. Streptavidin (0.1 mg mL^−1^ in PBS) was subsequently flowed across the surface to an immobilization level of 2000 RU. Ethanolamine (GE Healthcare; 1M, pH = 8.5; 10 µL min^−1^ for 7 min) was used to block any unreacted activated esters. CXCR4 ACMs (0.01 mg mL^−1^ in running buffer) were then immobilized at 5 µL min^−1^ to the desired immobilizations level. BSA (5 mg mL^−1^) was added to the running buffer to reduce nonspecific binding.

CXCR4 VLPs (Integral Molecular, PA) were captured by direct amine-coupling to the carboxylic acid-functional surface. The VLPs (1∶100 dilution of 400 units in PBS) were then flowed across the surface, resulting in the immobilization of the VLPs. Immobilization level was 5000 RU. Active ester groups were blocked with ethanolamine (1 M, pH = 8.5).

### Binding analysis

For analysis, the indicated concentrations of CXCR4 conformational antibody (CD184, BD Pharmigen, NJ) in running buffer were flowed across the surface for 100 s at 70 µL min^−1^ in sequence of increasing concentration with a single final dissociation phase of 10 min. As negative controls, blank polymersomes were used that had been subjected to the in-vitro synthesis procedure in the absence of c-DNA (null particles in the case of VLPs). Data presented is double-referenced against running buffer and reference surface. Binding analysis was performed using the supplied software (Bia-evaluation for T-200).

## Results and Discussion

**Figure 1:**
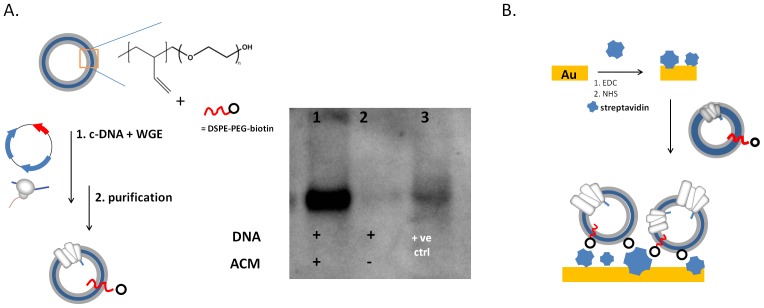
In-vitro synthesis and direct insertion of CXCR4 into polymersomes. A: PB-PEO polymersomes and CXCR4 c-DNA were subjected to in-vitro synthesis using a wheat germ coupled translation-transcription extract (WGE) and purified by a filtration step. After filtration, insertion of CXCR4 in the polymersome membrane was verified by Western blot. CXCR4 c-DNA in absence of polymersomes, which went through the same process, did not show the presence of CXCR4. The positive control is a commercially available CXCR4 cell membrane preparation. B: After purification, ACMs were immobilized onto a biosensor gold chip by first coupling streptavidin using standard EDC/NHS coupling, and then capturing the CXCR4 ACMs by the interaction with streptavidin of biotinylated lipid mixed into the polymersome membrane.

We initially set out to express CXCR4 according to our previously published procedures, i.e., expression of the protein using a coupled transcription-translation wheat germ extract (WGE; Figure 1A). [12] For immobilization on gold chips, it could be argued that the commonly employed amine-coupling chemistry may lead to the immobilization of some unfolded receptors in the form of aggregates, whether or not stabilized by components of the wheat germ extract. In order to rule out the occurrence of these possibilities, we modified our experimental procedures. First, we adapted the purification protocol of ACMs to exclude any unincorporated/free CXCR4 from solution. Using this method, a clear CXCR4 band in the western blot was seen only when ACMs were present in the in-vitro synthesis (IVS) reaction (Figure 1A). Second, we adapted the surface immobilization method in SPR to show that conformational binding *solely* originated from CXCR4 receptor inserted into the polymersome membrane. Henceforth, we coupled streptavidin to the gold chip by amine coupling, and captured the CXCR4-ACMs by interacting with a small fraction (1%) of biotinylated lipids (1,2-distearoyl-*sn*-glycero-3-phosphoethanolamine-*N*-[biotinyl(polyethylene glycol)-2000 (DSPE-PEG-biotin) that was mixed in with the polymersome membrane (Figure 1B). As a result, CXCR4-ACMs were stably immobilized on the biacore chip, presenting only receptors integrated in the polymer membrane.

**Figure 2:**
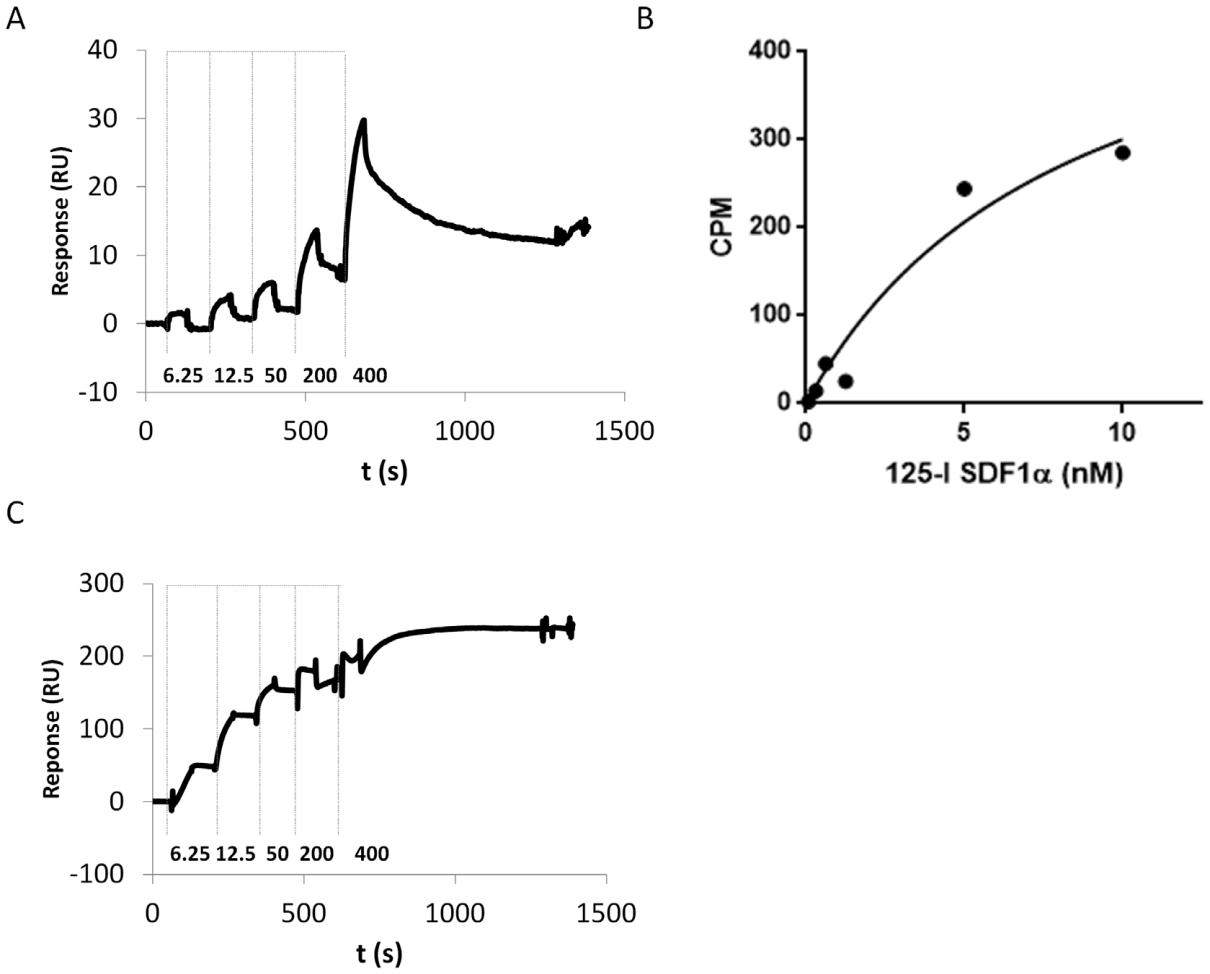
Kinetic screening of 12G5 mAb binding to CXCR4-ACMs immobilized onto biosensor chips. A: Ab was injected at increasing concentrations (6.25–400 nM) over 100 s, followed by a buffer wash (without regeneration) between injections (immobilization level: ca. 5000 RU; biotin/streptavidin immobilization). B. Saturation binding of 125-I SDF1α to CXCR4-ACMs. A dissociation constant of 8.4 nM was determined. C. The same series of measurements as shown in Fig. 2 A, conducted using immobilized VLPS (immobilization level: 5000 RU).

Following this approach we immobilized the C4-ACMs at immobilization levels of ca. 5000 RU and evaluated the binding of the monoclonal antibody (mAb) 12G5 to the receptor by running a concentration series of increasing concentration over the receptor surface (Figure 2). Here it should be noted that the ACMs display diameters from 150 to 200 nm such that the greater proportion of the membranes is within the evanescent regime. [12], [18] The mAb 12G5, directed against CXCR4, recognizes a conformation-dependent epitope involving the second and third extracellular domains (ECL1 and ECL2) of CXCR4, as well as the N-terminal domain. [19] Therefore, binding of 12G5 to CXCR4-ACMs would indicate the presence of the correctly folded receptor, oriented with the extracellular domain facing the outside solution. For comparison, we employed commercially available virus-like particles (VLPs; particles that are derived from cell membranes and carry enriched receptor) presenting CXCR4. CXCR4 proteoliposomes (supplier) (structurally similar to ACMs but having a lipid bilayer membrane) gave a relatively small signal during initial testing in our hands (data not shown) so that we decided to pursue our studies using CXCR4 VLPs as a comparison. Both VLP and proteoliposome preparations have been shown to bind ligands, with CXCR4 VLPs having been successfully used in biosensor analysis. [20], [21], [22].

Using CXCR4-ACMs, we observed a concentration-dependent increase in response, which fitted well to a 1∶1 binding interaction and exhibiting clear association and dissociation phases between injections. We did not observe more complex kinetics resulting from bivalent binding. The shape of the sensorgrams, being linear rather than exponential especially at higher mAb concentrations, indicated the occurrence of mass transfer such that, at the current immobilization levels, receptor concentration was probably too high. Nevertheless, these experiments indicate that the mAbs bound readily to CXCR4-ACMs, signifying two important facts: (1) in-vitro CXCR4 receptor inserted into polymersome membranes retain their native conformation of the extracellular side, and (2) the receptor is present in appreciable concentrations with the extracellular side facing the bulk solution.

To further assess the functionality of the receptor incorporated in ACMs, we performed additional experiments to study binding of radiolabeled (I-125) SDF-1α (stromal cell-derived factor-1 alpha, the natural ligand of CXCR4) to CXCR4 in ACMs and native membrane preparations (Figure 2B; See supporting information for details). Dissociation constants values for SDF-1α were found to be 8.4 nM and 1.4 nM for CXCR4-ACMs and CXCR4 membrane preparations, respectively (Figure S1 and S2). Combined with the data of binding of conformationally-sensitive Ab, these results show that CXCR4 embedded in polymersome membranes binds ligands and Abs with affinities comparable with native protein in natural lipid membranes. Apart from the difference in properties between the polymer membrane and the native membrane, the somewhat lower affinity of SDF-1α for ACMs may result from the fact that in this synthetic system G proteins are absent. These proteins are known to affect the receptor’s conformation and may therefore alter ligand-binding affinity. [23].

For CXCR4-VLPs, we immobilized the VLPs by standard amine-coupling, as reported previously. [20] Even though the approach may not necessarily be optimal for analyzing VLP-ligand interactions by biosensor analysis, this still allowed us to assess the performance of our approach versus other receptor preparations. 12G5 binding to VLPs gave good binding responses initially, with clear association curves. Dissociation was, however, subdued pointing to accumulation of material on the chip surface and hampering further screening. This phenomenon is in line with the previously reported observation that virus-receptor interactions are multivalent and generally irreversible. [18] Therefore, these preparations seem more amenable to a sandwich set-up where the receptor-presenting particles are captured by an Ab-covered surface, after which the ligand of interest is injected. After binding, the surface is regenerated to expose the first Ab and the cycle is repeated. Such an approach prevents reproducibility issues caused by rapid deactivation of the receptor preparation when reconstituted via traditional approaches (detergent, lipid). Indeed, a similar approach has been shown to be successful for SPR-based binding analysis of crude CXCR4 cell membrane preparations. [14], [15].

**Figure 3:**
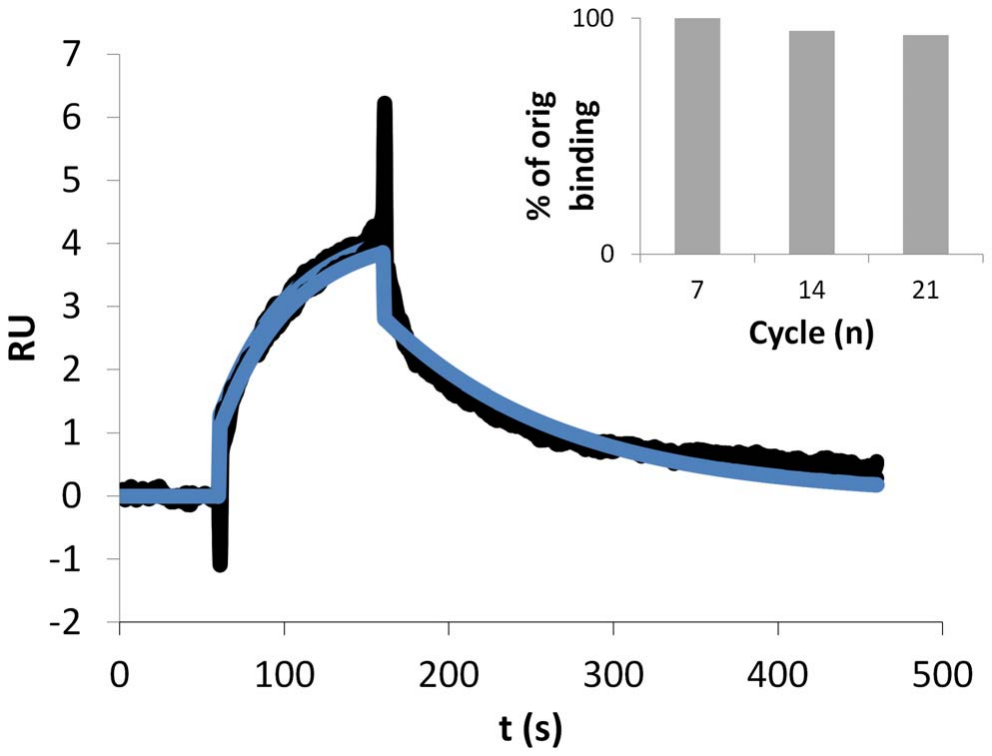
Sensorgrams of mAb 12G5 binding (100 nM) to CXCR4-ACMs immobilized at low RU (ca. 1400) via biotin/spteptavidin immobilization of the embedding polymersome matrix. The analyte was injected in triplicate, at cycle 7, 14, and 21. Intermediate cycles involved blank injections. The blue line shows the fit to the curve assuming 1∶1 binding kinetics. The inset shows the relative decrease in binding activity of the surface as measured by the binding level 4 s before the end of the injection.

Having demonstrated the presence of properly folded receptor in ACMs, the receptor preparation was subjected to repeated injection cycles of CXCR4 mAb. To prevent mass-transfer from occurring, CXCR4-ACMs were immobilized at lower immobilization levels (∼1500 RU on the sensor chip), and the immobilized receptor surface was subjected to multiple injections of 12G5 (100 nM), over a total of 25 injections lasting a total of 4 hours. At these immobilization levels, although binding levels were much reduced, a good quality of binding curves was obtained as exemplified by association phases that showed clear exponential binding behaviour and almost full dissociation (Figure 3). Moreover, the curves fitted well to a 1∶1 binding model. Interestingly, repeated cycles of conformational antibody injection demonstrated an unprecedented conformational stability of the receptor preparations under the analysis conditions: over the time span of 24 cycles (ca 4 h), only a minimal decrease (7%) in the activity of the receptor surface was observed. Given the stability of polymersomes preparation in general, [11], [24] the cause for the eventual, albeit minor, degradation of the signal is most likely protein-related and may either be attributed to loss of ACMs from the surface or unfolding of the receptor. Since there was no correlation of the decreasing binding signal to the drop in baseline (which showed an initial drop, after which it stabilized (data not shown)), and assuming that the decrease in baseline points to loss of material from the sensor surface, the decrease in activity of the receptor comes from degradation of the receptor itself, most likely unfolding.

**Figure 4:**
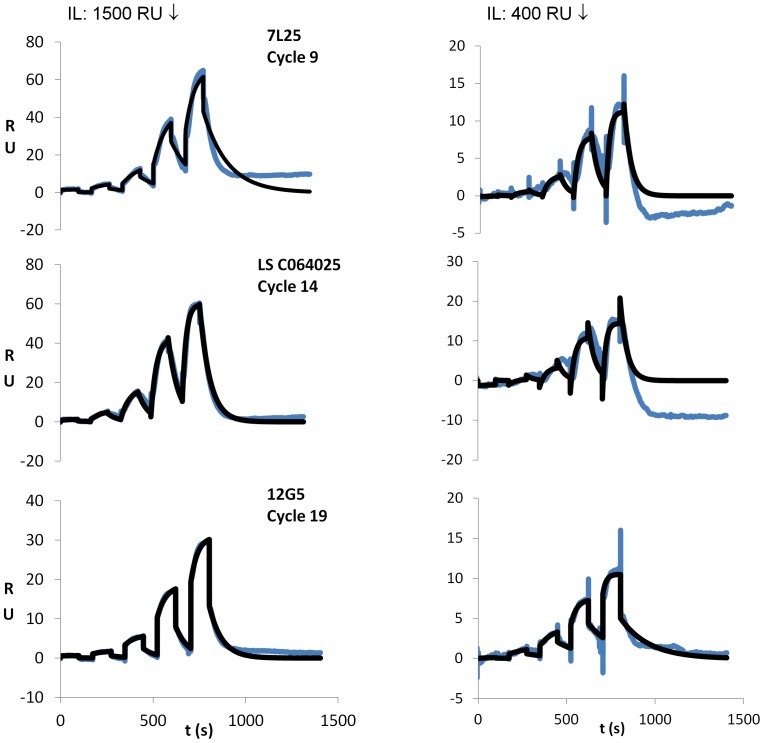
SPR sensorgrams obtained by kinetic screening of three different types of conformationally sensitive ABs to a single CXCR4 ACM preparation immobilized on a sensor chip. Conditions were the same as in Figure 2, except for the immobilization level, which is as indicated in the figure.

To demonstrate how the stabilized native CXCR4 could be employed, a small screen was conducted with, apart from 12G5, two additional monoclonal antibodies against CXCR4, i.e. clone 7L25 and C064025, a monoclonal IgG2B antibody. The three mAbs were screened versus the same CXCR4-ACM preparation, separately injecting each antibody in a series of increasing concentration (for kinetic analysis) (Fig. 4). The 12G5 antibody was screened last in the sequence to compare the binding parameters obtained with that obtained in Figure 3. Testament to the conformational stability of the receptor preparation, all binding curves exhibited clear association and dissociation phases, irrespective of the receptor density or cycle number of the injection, once again confirming the presence of a stable, properly folded receptor in the polymer membrane. Generally, the fits to the curves at 400 RU were more accurate than that at 1500 RU, which we attribute to the fact that, at lower receptor densities, the fit of concentration with binding level all saturated. From the fits at 400 RU, the detailed binding parameters were extracted (*k*_on_, *k*_off_, and K_D_), as summarized in Table 1. Comparison of the binding parameters for the 12G5 mAb yielded values of 69 (+/−) nM and 52 nM for the 100 nM screen and kinetic titration, respectively, indicating that the developed protocol was reproducible. Here some caution is warranted because the values were obtained at different immobilization levels (1500 vs 400 RU), and the fit to the sensorgram at 1500 RU involved only a single (albeit triplicate) concentration. The values for the other two antibodies were somewhat higher with roughly similar *k*_on_ and *k*_off_ values. The results indicate that 12G5 is somewhat stronger than binding of 7L25 and C064025, although the variation in either of the binding parameters (*k*_on_, *k*_off_, and K_D_) was not that large. This appears to be in line with earlier observations on screening of CXCR4-specific antibodies where it was observed that most antibodies exhibited fairly similar affinities (in this instance, EC 50 values). [25].

**Table 1:** Kinetic parameters extracted from fitting the binding curves of concentration series of three different mAbs against a single preparation of immobilized CXCR4 ACMs.

	*k*_on_ (×10^3^ Ms^−1^)	*k*_off_ (×10^−3^ s^−1^)	K_D_ (nM)
12G5[a]	120	6.3	52.0
C064025	91.1	25.5	280
7L25	78.7	25.3	322

[a] KD for 12G5 binding to CXCR4-ACMS shown in Figure 2 was 60.2±17 nM.

The biosensor data demonstrate that CXCR4 embedded via cell-free expression in polymeric membranes possess an on-chip stability that is at par, or even enhanced, as compared to receptors expressed by cell-based expression and subjected to detergent-assisted lipid reconstitution. [16] Importantly, the binding data observed are commensurate with that observed for cell-based CXCR4 expression (as observed for binding of SDF-1α, suggesting that merely the presence of an amphiphilic membrane is able to provide the stable integration of (complex) membrane proteins. Concerning the physical stability of the preparation (as judged from its amenability for extended biosensor interrogation) we expect the polymer-based bilayer membrane to provide support for the CXCR4 by two independent mechanisms that result from the enhanced physical stability of bilayer polymer membranes. First, the polymer membrane can withstand the demanding physical restraints (high concentration of solutes, osmolarity) of direct incubation with the cell-free extracts and remains intact during purification. Second, the bilayer polymer membrane is sufficiently stable to allow its intact surface immobilization, providing a stable matrix for multiple-hour interrogation of the binding characteristics of the membrane protein by surface-plasmon resonance. The approach therefore allows to circumvent the deleterious effect that detergent-solubilization may have on receptor structure through direct embedding in the more native environment of an amphiphilic membrane. Evidently, one topic of future investigation should be how the approach may benefit the biophysical characterization of other members of the GPCR family, especially when it comes to poorly characterized (i.e., fragile) receptors such as olfactory receptors, which at the same time suffer from low expression levels, where we argue that the outcome would mainly be dependent on the properties of the receptor itself, as both the IVS and biosensor characterization protocol is expected to be readily applicable to the GPCR family, since it is essentially only the DNA sequence that changes. Indeed, it is encouraging that the expression protocol we employed is essentially the same as what we reported for the (unrelated) dopamine receptor D2. [12].

To gauge, nevertheless, the stabilization of CXCR4 embedded in the polymer bilayer as compared to detergent-solubilized receptor, we applied the developed protocol to measurement of the melting temperature of the receptor by label-free biosensing and tested both CXCR4-ACMs and detergent (P20) solubilized receptors, expressed by IVS (See SI for a detailed protocol). Although this detergent may not be optimal for receptor-stabilization, it was compatible with the IVS extract, whereas milder detergents such as CHAPS were not. As expected, binding levels observed for the detergent-solubilized receptor were low, but did give rise to a measurable signal, which after normalization could be compared to the curve obtained for ACMs (Figure S3). For detergent-solubilized CXCR4 and CXCR4-ACMs, melting temperatures were obtained of 29.7 and 38.8°C, respectively, demonstrating that the polymer bilayer shifts the melting temperature of the protein fold by almost 10°C. Hence, the data supports our hypothesis that direct expression into native-like bilayer membranes stabilizes the receptor when compared to a process whereby the heterologously expressed receptor has to go through a detergent-solubilization step.

In summary, the results show that native CXCR4 receptor produced by in-vitro expression and inserted in polymer membranes can serve as an alternative to currently available methods of cell-based production, receptor engineering and complex reconstitution procedures, with the main advantage being that the receptor can be produced in sufficient quantity in a manner of hours. We are currently exploring the generality of the developed approach to include other members of the GPCR family.

## Supporting Information

Figure S1 **Displacement of radio-labeled SDF1-a using CXCR4 cell membrane preparations.** Radioligand binding experiments were conducted at GVK Biosciences, Hyderabad, India. CxCR4 ligand binding studies were performed in 96-well plate in a total volume of 200 µL consisting of 50 µL of 125I-SDF, 50 µL of assay buffer (50 mM HEPES pH 7.4, 5 mM MgCl2, 1 mM CaCl2, 0.2% BSA) and 100 µL of membrane preparation diluted in appropriate buffer. Non-specific binding was determined in the presence of 10 µM of unlabeled SDF or untransfected control membrane. The plate was incubated at room temperature for 2 h. Reactions were terminated by flash filtration and inverse transfer to 96-well glass fibre filter plates. The plate was then dried for 30 minutes at 60°C and sealed at the bottom with an adhesive sheet. Subsequently, 50 µL of scintillation fluid was added to each well, the plates sealed on top and the radioactivity counted in a 96-well plate counter (Top count NXT, Perkin Elmer). The assay was first validated using commercially available CXCR4 membrane preparations. A fixed concentration of radiolabeled 125I-SDF (0.5 nM) was used to determine the IC50 by incubating the CXCR4 membrane with different concentration of unlabeled SDF. Linear regression was performed using Graphpad Prism. The IC50 value was 1.4 nM, correlating with the value stated by the supplier (0.9 nM).
(TIF)

Figure S2 **Saturation binding of 125-I SDF1α to CXCR4-ACMs.** For CXCR4 ACMs, first the optimal concentration was determined with respect to specific binding. At 0.625 µg/well, the TB/NSB ratio measured 3.8 and the percentage specific binding was 74% (data not shown). The CXCR4 ACMs were then subjected to a saturation assay (constant receptor concentration, varying ligand concentration), with and without 0.5 µM unlabeled SDF. The dissociation rate constant, *K*_d_, and the maximal number of receptor binding sites, *B*_max_, was calculated using GraphPad Prism.
(TIF)

Figure S3 **Melting curves of CXCR4 ACMs and CXCR4 solubilized in detergent.** CXCR4 ACMs were freshly prepared as described above and kept at 4°C. For IVS of CXCR4 in P20 solution, the same protocol was followed but instead of ACMs, detergent was added to a concentration of 1%. Although the detergent selected may not be optimal for receptor-stabilization, it was compatible with the IVS extract, whereas milder detergents such as CHAPS were not. Expression was verified by Western blot after removal of the insoluble fraction by centrifugation. To preserve activity, the resulting detergent solubilized receptor was used on the same day without further purification. For determination of the melting temperature, samples were incubated for 15 minutes in a PCR thermal cycler at temperature increments of 10°C, from 10–80°C. After incubation the samples were cooled to 4°C and directly analyzed for their binding activity. For binding analysis, 12G5 mAB to CXCR4 was immobilized to the custom-made chip essentially as detailed for immobilization of streptavidin, upon which the CXCR4 preparations (ACM or detergent) were injected. Temperature data was analyzed using GraphPad Prism. Data is represented in percentage of initial response to correct for the differences in activity of CXR4 ACMs and detergent-solubilized receptor.
(TIF)

Text S1 **Materials and methods used in the experiments.**
(DOCX)
