# Supplementary figures and images for: Conformational Antibody Binding to a Native, Cell-Free Expressed GPCR in Block Copolymer Membranes
Source: PLoS One. 2014 Oct 20;9(10):e110847. doi: 10.1371/journal.pone.0110847 (PMC4203850; doi:10.1371/journal.pone.0110847)

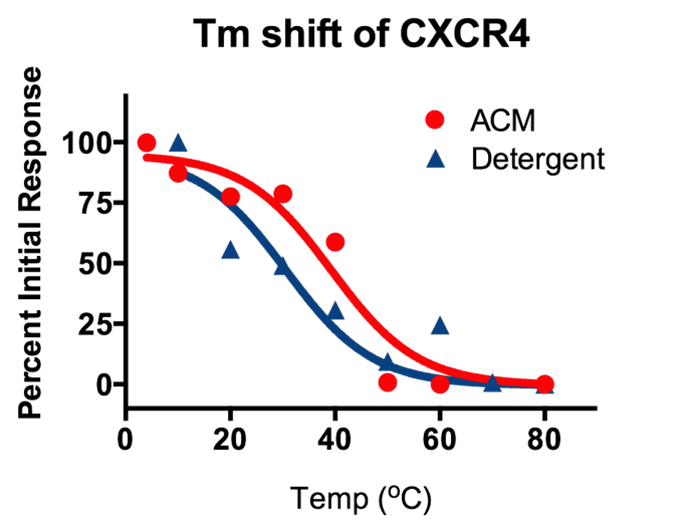

Supplement: Figure S3 — Melting curves of CXCR4 ACMs and CXCR4 solubilized in detergent. CXCR4 ACMs were freshly prepared as described above and kept at 4°C. For IVS of CXCR4 in P20 solution, the same protocol was followed but instead of ACMs, detergent was added to a concentration of 1%. Although the detergent selected may not be optimal for receptor-stabilization, it was compatible with the IVS extract, whereas milder detergents such as CHAPS were not. Expression was verified by Western blot after removal of the insoluble fraction by centrifugation. To preserve activity, the resulting detergent solubilized receptor was used on the same day without further purification. For determination of the melting temperature, samples were incubated for 15 minutes in a PCR thermal cycler at temperature increments of 10°C, from 10–80°C. After incubation the samples were cooled to 4°C and directly analyzed for their binding activity. For binding analysis, 12G5 mAB to CXCR4 was immobilized to the custom-made chip essentially as detailed for immobilization of streptavidin, upon which the CXCR4 preparations (ACM or detergent) were injected. Temperature data was analyzed using GraphPad Prism. Data is represented in percentage of initial response to correct for the differences in activity of CXR4 ACMs and detergent-solubilized receptor. (TIF) [file pone.0110847.s003.tif]
