# Supplementary material for: Conformational Antibody Binding to a Native, Cell-Free Expressed GPCR in Block Copolymer Membranes
Source: PLoS One. 2014 Oct 20;9(10):e110847. doi: 10.1371/journal.pone.0110847 (PMC4203850; doi:10.1371/journal.pone.0110847)
Supplement: Text S1 — Materials and methods used in the experiments. (DOCX) [file pone.0110847.s004.docx]

Materials and methods used in the experiments

**Materials**

Bare biosensor Au chips, 11-mercapto-undecanoic acid, 1-ethyl-3-(3-dimethylaminopropyl)carbodiimide, *N*-hydroxysuccinimide, and ethanolamine were from GE Healthcare, UK. CXCR4 lipoparticles were obtained from Integral Molecular (Philadelphia, PA) and Chemiscreen^TM^ CXCR4 receptor membrane preparations were from Perkin Elmer (Waltham, MA) CXCR4 conformational antibody 12G5 was from BD Pharmingen (East Rutherford, NJ), while C064025 was from LifeSpan BioSciences Inc (Seattle, WA) and 7L25 from Novus Biologicals (Littleton, CO) TNT T7 coupled wheat germ extract system was from Promega (Madison, WI). PEG antibody was from Abcam, SDF1α and CXCR4 antibody for Western blot were from Abcam (Cambridge, UK). All other reagents were from Sigma Aldrich (St Louis, MO).

Polymersomes were prepared as reported and purified by dialysis (MWCO 50 kDa, Spectra/Por® 7, Spectrum Laboratories) against ultrapure water [1].

**Cloning of CXCR4**

Chemokine receptor CXCR4 gene (Source Bioscience Lifescience: OmicsLink ORF Expression Clone: A0740; Human BAC (RPCI-11) -1; transcript variant 1 (long); LOCUS NM_000795) was cloned into the pCMVTNT vector according to standard molecular biology protocols. The gene for CXCR4 was amplified with polymerase chain reaction (PCR) from the pCMVTNT-CXCR4-GFP2 plasmid using the primers (forward: 5'-CCGGAATTCTTTTTTTTTTAAACCACCATGGATCCACTGAATCTGTCC-3'; reverse: 5'-GCCGCGGCCGCTTAGCAGTGGAGGATCTTCAGG-3' 1st base). In this step, an EcoRI restriction site and a Kozak sequence were introduced into the pCMVTNT vector before the gene. The PCR fragments and pCMVTNT vector were then digested with EcoRI and NotI followed by a ligation reaction. The cloning was veriﬁed by sequencing analysis.

**In vitro expression of CXCR4**

For cell free in-vitro expression of DRD2 a TNT T7 coupled wheat germ extract system was used. Briefly, a 50 µL reaction mixture containing 2.0 µg CXCR4 DNA template was prepared according to the supplier’s protocol and incubated for 20 h at 15 °C. As a control, the same reaction mixture without cDNA or native CXCR4 membrane preparations was used. Successful expression was determined by Western Blot analysis. After in vitro expression of the protein the proteopolymersomes were purified from the in vitro synthesis mixture using Durapore PVDF 1000 k MWCO centrifugal ﬁlters (Millipore) retaining only proteins associated with the polymersomes.

**Western blot**

5 µL of in vitro reaction mixture was used for gel electrophoresis (NuPAGE 10% Bis-Tris Gel, MES running buffer, Invitrogen, 200V, 30 min) and Western Blot (iBlot system with PVDF membrane and Western Breeze® Chemiluminescence Kit, anti-mouse, Invitrogen) according to the supplier’s manual. The blot was incubated overnight in a 1:1000 dilution of anti-CXCR4.

[1] M. Nallani, M. Andreasson-Ochsner, C. W. Tan, E. K. Sinner, Y. Wisantoso, S. Geifman-Shochat, W. Hunziker, Biointerphases 2011, 6, 153.
